# Education and electronic health record implementation–better together? An assessment of compliance of oral vancomycin dosing for *Clostridioides difficile* infection and the impact of different antimicrobial stewardship interventions

**DOI:** 10.1017/ash.2025.10272

**Published:** 2026-01-13

**Authors:** Jordan Chiasson, Jeremie Sawadogo, Michael Kent

**Affiliations:** 1 Department of Clinical Pharmacy, JPS Health Network, Fort Worth, TX, USA; 2 Department of Pharmacotherapy, UNT System College of Pharmacy, University of North Texas Health Science Center, Fort Worth, TX, USA

## Introduction

Educational initiatives are vital to improving antimicrobial prescribing, but they are typically time intensive, requiring recurring sessions to ensure appropriate uptake. Leveraging technology into antimicrobial stewardship (AMS) interventions is currently recommended by national guidelines.^1,2^ A multistep approach utilizing both educational and technological initiatives may be beneficial, leading to sustained outcomes. In this quality assessment, we aimed to examine the impact of education followed by an electronic health record (EHR) imbedded intervention.

## Methods

### Rationale and interventions

This project was completed as part of a hospital wide AMS quality assessment project in multiple stages at a large county hospital in Fort Worth, Texas.

Local institutional Clostridioides difficile infection (CDI) guidance, which included diagnostic and treatment recommendations, was updated March 2021 based on available national guidelines.^3^ Educational sessions were provided to various service lines along with a guidance document on the institution’s AMS intranet page. Recommended dosing for oral vancomycin included 125 mg four times daily (non-severe and severe infections) or 500 mg four times daily (fulminant infections). Through pharmacist verification, several instances of vancomycin 250 mg daily were noted to have been ordered after educational sessions. Additional direct education on local clinical guidance was provided to ordering clinicians in these instances.

In August 2022, local formulary change resulted in the preferred dosing form of oral vancomycin changing from oral solution to capsules. Local leadership tasked the AMS team with reviewing an updated EHR drug file entry for oral vancomycin. The AMS team recommended removing the 250 mg dosage button for order entry. Providers could type in 250 mg dosages if desired, but only buttons for 125 mg and 500 mg remained.

Fifteen months post implementation of the new drug file, a quality assessment was initiated to review and determine if further interventions were necessary to improve prescribing patterns. An estimated 10 hours of pharmacist time was spent on implementation.

### Electronic health record (EHR) data extraction and analysis

Inpatient pharmacy dispense workload data regarding oral vancomycin from December 1, 2019, to November 31, 2023, was extracted from the EHR (Epic Slicer Dicer). Three time periods were assessed.

- Period 1: Preintervention (December 2019 – February 2021)
  - ◦ March 2021 – May 2021 was excluded as a 3-month washout for dissemination of updated local guidance.
- Period 2: Postimplementation of Local Guidance (June 2021 – August 2022)
- Period 3: PostEHR Update (September 2022 – November 2023).

No patient level data was reviewed in the analysis. Monthly data for oral vancomycin orders and administration were separated by dose (125 mg, 250 mg, and 500 mg). Monthly data was compiled for each period. Poisson regression models were run to compare Period 1 to Periods 2 and 3 using the number of inappropriate orders and administrations of oral vancomycin as outcomes and the three different periods as predictors. All analyses were performed with the use of SAS (version 9.4).

### End points

The primary end point was the number of administrations of oral vancomycin 250 mg doses during each period. Secondary endpoints included the number of orders of oral vancomycin 250 mg during each period and the percent of inappropriate administrations and orders. Orders and administrations were defined as inappropriate if the dose was 250 mg.

## Results

During the 45 months of data reviewed, 1,200 orders for oral vancomycin were placed with 17,248 administrations documented. Inappropriate administrations and orders decreased in each subsequent period. Full data for all time periods are presented in Table 1.

**Table 1.** Orders and administrations of oral vancomycin by study period

Period^*^	Total administrations, *n*	Inappropriate administrations, *n* (%)	Total orders, *n*	Inappropriate orders, *n* (%)
Preintervention (Period 1)	5,618	253 (4.5)	407	29 (7.13)
Postimplementation (Period 2)	6,531	155 (2.37)	413	20 (4.84)
PostEHR Update (Period 3)	5,099	9 (.18)	380	2 (.53)

* Each period comprised 15 months.

EHR: electronic health record.

The estimated Poisson regression coefficients and corresponding test statistics for the predictor period comparing periods were as follows:

For the number of inappropriate administrations: Period 1 to Period 2 showed a decrease by .49 (23.07 [χ^2^]; *P* value < .0001), and Period 1 to Period 3 showed a decrease by 1.6681 in the number of inappropriate administrations (96.73 [χ^2^]; *P* value < .0001).

For the number of inappropriate orders: Period 1 to Period 2 showed a decrease by .3716 (1.63 [χ^2^]; *P* value = .2011), and Period 1 to Period 3 showed a decrease by 1.3371 (13.38 [χ^2^]; *P* value = .0003).

## Discussion

This multistep quality assessment initiative demonstrated an impact on oral vancomycin prescribing at a single institution utilizing both educational and technological interventions. The Poisson regression analysis revealed a statistically significant decrease in both inappropriate administrations and orders comparing Period 1 to Period 3, as well as for inappropriate orders from Period 1 to Period 3, but did not show a statistically significant decrease for inappropriate orders from Period 1 to Period 2 (at alpha = .05) for the estimated coefficients of the predictor period.

Clinician facing EHR medication ordering processes should be reviewed regularly for opportunities to support providers in ordering safe and effective doses of preferred agents. This review of vancomycin ordering was prompted by a logistical change but afforded an opportunity for an AMS intervention. Review of ordering processes within the EHR should be incorporated into action plans of AMS initiatives.

Limitations of the present study include that this is a single health system initiative performed retrospectively as a quality improvement project, thus no control arm was available. The data was extracted from the EHR without patient level review so there was no validation of appropriateness of CDI treatment or patient level outcomes.

This example of a real-world AMS initiative over multiple years shows the impact of a multifactorial approach; integrating technology, updating local guidance, and providing education to optimize prescribing. Through this approach inappropriate prescribing of oral vancomycin was reduced and remained low for a sustained period.
